# Supplementary material for: Estimated incidence and case fatality rate of traumatic brain injury among children (0–18 years) in Sub-Saharan Africa. A systematic review and meta-analysis
Source: PLoS One. 2021 Dec 30;16(12):e0261831. doi: 10.1371/journal.pone.0261831 (PMC8717989; doi:10.1371/journal.pone.0261831)
Supplement: S2 Table — (DOCX) [file pone.0261831.s002.docx]

**S2 Table:** Search strategy for PubMed, Google scholar and AJOL

| Search # | Search term |
| --- | --- |
|  | Children OR pediatric OR childhood |
|  | ’traumatic brain injury’’ OR ‘’traumatic head injury’’ |
|  | 1 AND 2 |
|  | ’mortality rate’’ OR ‘’death rate’’ |
|  | 3 AND 4 |
|  | incidence OR burden OR Prevalence |
|  | 5 OR 6 |
|  | sub-Saharan Africa |
|  | Angola OR Benin OR Botswana OR Burkina Faso OR Burundi OR Cameroon OR Cape Verde OR Central African Republic OR Chad OR Comoros OR Congo OR Cote d'Ivoire OR Djibouti OR Equatorial Guinea OR Ethiopia OR Gabon OR The Gambia OR Ghana OR Guinea OR Guinea-Bissau OR Kenya OR Lesotho OR Liberia OR Madagascar OR Malawi OR Mali OR Mauritania OR Mauritius OR Mozambique OR Namibia OR Niger OR Nigeria OR Rwanda OR Sao Tome and Principe OR Senegal OR Seychelles OR Sierra Leone OR Somalia OR South Africa OR Sudan OR Swaziland OR Tanzania OR Togo OR Uganda OR Zaire OR Zambia OR Zimbabwe |
|  | 8 OR 9 |
|  | Limit to January, 2010-December,2020 |
|  | Limit to Humans |
|  | 10 AND 11 AND 12 |
