# Supplementary material for: Estimated incidence and case fatality rate of traumatic brain injury among children (0–18 years) in Sub-Saharan Africa. A systematic review and meta-analysis
Source: PLoS One. 2021 Dec 30;16(12):e0261831. doi: 10.1371/journal.pone.0261831 (PMC8717989; doi:10.1371/journal.pone.0261831)
Supplement: S3 Table — (DOCX) [file pone.0261831.s003.docx]

**S3 Table**: Risk of bias/quality assessment

| Study | Year | Methodological quality | Comparability | Outcome assessment | Score | Remark |
| --- | --- | --- | --- | --- | --- | --- |
| Abdelgadir et al., (4) | 2017 | ******* | ****** | ******* | **8** | Low risk |
| Vaca et al (15) | 2019 | ******** | ***** | ****** | **7** | Low risk |
| Punchak et al., (13) | 2018 | ******* |  | ******* | **6** | Moderate risk |
| Schrieff et al., (24) | 2013 | ******* |  | ***** | **4** | High risk |
| Bedry et al (16) | 2020 | ******** | ****** | ******* | **9** | Low risk |
| Udoh et al (25) | 2013 | ******** |  | ****** | **6** | Moderate risk |
| Buitendag et al., (19) | 2017 | ******** |  | ****** | **6** | Moderate risk |
| Okyere-Dede et al (23) | 2013 | ******** |  | ****** | **6** | Moderate risk |
| Lalloo et al (21) | 2004 | ******** |  | ****** | **6** | Moderate risk |
| Egbonhou et al., (17) | 2019 | ******* |  | ****** | **5** | Moderate risk |
| Hode et al., (18) | 2016 | ******** |  | ****** | **6** | Moderate risk |
| Kouitcheu et al (20) | 2020 | ******** |  | ****** | **6** | Moderate risk |
| Mendy et al (22) | 2014 | ******** |  | ****** | **6** | Moderate risk |
