# Supplementary material for: Estimated incidence and case fatality rate of traumatic brain injury among children (0–18 years) in Sub-Saharan Africa. A systematic review and meta-analysis
Source: PLoS One. 2021 Dec 30;16(12):e0261831. doi: 10.1371/journal.pone.0261831 (PMC8717989; doi:10.1371/journal.pone.0261831)
Supplement: S4 Table — (DOCX) [file pone.0261831.s004.docx]

**S4 Table:** Sensitivity analysis of pooled case fatality rate for each study being omitted with 95% CI

| Study omitted | Pooled case fatality Rate (%) | 95% CI |
| --- | --- | --- |
| Abdelgadir et al., | 8.0 | 3.0-14.0 |
| Vaca et al | 8.0 | 3.0-14.0 |
| Punchak et al., | 9.0 | 3.0-14.0 |
| Schrieff et al., | 8.0 | 3.0-13.0 |
| Bedry et al | 9.0 | 4.0-14.0 |
| Udoh et al | 8.0 | 3.0-13.0 |
| Buitendag et al., | 9.0 | 4.0-14.0 |
| Okyere-Dede et al | 9.0 | 4.0-14.0 |
| Lalloo et al | 9.0 | 5.0-14.0 |
| Egbonhou et al., | 7.0 | 2.0-11.0 |
| Hode et al., | 9.0 | 5.0-14.0 |
| Kouitcheu et al | 8.0 | 3.0-13.0 |
| Mendy et al | 8.0 | 3.0-13.0 |
| Overall | 8.0 | 3.0-13.0 |
