# Supplementary material for: Estimated incidence and case fatality rate of traumatic brain injury among children (0–18 years) in Sub-Saharan Africa. A systematic review and meta-analysis
Source: PLoS One. 2021 Dec 30;16(12):e0261831. doi: 10.1371/journal.pone.0261831 (PMC8717989; doi:10.1371/journal.pone.0261831)
Supplement: Attachment — Submitted filename: Response to reviewers.docx [file pone.0261831.s006.docx]

**Response to reviewers’ comments**

I sincerely express my warmest greetings to you and your prestigious journal for your comments and feedback. I write on behalf of my co-authors to submit our reply to your astute experienced reviewers' insightful comments. The methodology used follows a point-by-point approach to responding to all comments. Please see below for our response.

Reviewer #1:

**Comment:** Abstract, Results: “The current reviewed” should be “The current review”.

**Response:** Thanks for the correction. This has been rectified

**Comment:** Introduction, line 1: I would suggest to change “acquired brain injuries” to “acquired brain injury”

**Response:** Thanks for the correction. This has been rectified

**comment:** Results, page 8 at the bottom: The sentence “This also ranged from 5.1% in South Africa 29, and 41.2% in south Africa 26” reads a bit strange. It is strange that the extremes of the range are within one country (South Africa). On looking up the citation, they appear to even be from the same hospital, but over a different time period. I would suggest to either delete the entire sentence, to delete the specific mention of South Africa, or – if maintained – add an explanation.

**Response:** Thanks for the correction. The sentence has now been deleted.

Reviewer #3

**comment:** Abstract (Introduction section): "need for accurate" should be "need for an accurate"

**Response:** Thanks for the correction. This has been rectified

**comment:** page 5 (1st line): "as the most cause" should be "as the most common cause" and "in all age group" should be "in all age groups"

**response:** Thanks for the correction. This has been rectified

**comment:** page 5 (1st full paragraph): "need for accurate and" should be "need for an accurate and"

**response:** Thanks for the correction. This has been rectified

**comment:** page 8 (Study Selection): "inclusion criteria and included" should be "inclusion criteria and were included"

**response:** Thanks for the correction. This has been rectified

**comment:** Figure 1: in the authors' response to my comment about this Figure, they said the explanation for the reduction from 200 to 70 was due to duplicate records. Maybe there is just confusion in how to read their figure. Typically, these figures show how many articles are at each stage with an intermediate box that shows how many were excluded (and reasons for exclusion) at each stage. For example, their box for "Records after abstracts and title screened" has n=70 articles and the box to the right says that 40 were excluded which then yields the 30 articles in the next box. My original question had to do with the lack of boxes to the right for the 1st 2 boxes (articles identified through database and record after duplicate removed). So, did authors arrive at 200 articles from the original 820 after removing duplicates? If not, what were the reasons that reduced the number from 820 to 200? Similarly, what were the reasons for reducing the 200 articles to 70 articles?

**Response**: Authors acknowledge the relevance of the reviewer’s comment and as a result, revised Figure 1.

**Comment:** page 9 (2nd to last line): change "from 5.1% in South Africa, and 41.2% in south Africa" to "from 5.1% to 41.2%".

**Responses:** Thanks for the correction. The sentence has now been deleted as recommended by reviewer 1.

**Comment:** page 12: change "This approximately corroborate with" to "This approximately corroborates with", "not surprising as study" to "not surprising as the study", "TBI cases hence" to "TBI cases and hence", and "well-coordinated effort are" to "well-coordinated effort is"

**response:** Thanks for the corrections. These have been rectified

**comment:** page 13: change "The current results is similar" to "The current results are similar"

**comment:** page 13: I previously made a comment about the sex-differences in TBI - these are first mentioned in the Discussion. If authors feel this point is important enough to make in the Discussion, it should also be mentioned in the results.

**Response:** Thanks for the comment. The sex difference has been mentioned in the result section [Page 8]

Comment: page 14: change "fall predominant the cause" to "fall as the predominant cause" and remove "whooping" from the Conclusion section

**response:** Thanks for the corrections. These have been rectified
